# Changes in mammography screening in Ontario and Alberta following national guideline dissemination: an interrupted time series analysis

**DOI:** 10.12688/f1000research.55004.1

**Published:** 2021-10-15

**Authors:** Christine Fahim, Natasha Wiebe, Rosane Nisenbaum, Jemila S. Hamid, Joycelyne E. Ewusie, Marcello Tonelli, Paula Brauer, Elizabeth Shaw, Neil Bell, Dawn Stacey, Nathalie M. Holmes, Sharon E. Straus

**Affiliations:** 1 Knowledge Translation Program, Li Ka Shing Knowledge Institute, St. Michael’s Hospital, Toronto, Ontario, M5B 1T8, Canada; 2 Department of Medicine, University of Alberta, Edmonton, Alberta, T6G 2G3, Canada; 3 MAP Centre for Urban Health Solutions, Li Ka Shing Knowledge Institute, Toronto, Ontario, M5B 1T8, Canada; 4 Dalla Lana School of Public Health, University of Toronto, Toronto, Ontario, M5T 3M7, Canada; 5 Department of Mathematics and Statistics, University of Ottawa, Ottawa, Ontario, K1N 6N5, Canada; 6 Biostatistics Unit, Father Sean O’Sullivan Research Centre, Hamilton, Ontario, L8N 4A6, Canada; 7 Cumming School of Medicine, University of Calgary, Calgary, Alberta, T2N 1N4, Canada; 8 Department of Family Relations and Applied Nutrition, University of Guelph, Guelph, Ontario, N1G 2W1, Canada; 9 Department of Family Medicine, McMaster University, Hamilton, Ontario, L8S 4K1, Canada; 10 Department of Family Medicine, University of Alberta, Edmonton, Alberta, T6G 2R3, Canada; 11 Faculty of Health Sciences, University of Ottawa, Ottawa, Ontario, K1N 6N5, Canada; 12 Ottawa Hospital Research Institute, Ottawa, Ontario, K1H 8L6, Canada; 13 Public Health Agency of Canada, Ottawa, Ontario, K1A 0K9, Canada; 14 Department of Medicine, University of Toronto, Toronto, Ontario, M5S 1A8, Canada

**Keywords:** Breast Cancer, Mammography, Screening, Preventive Health Care, Knowledge Translation

## Abstract

**Background:** In November 2011, the Canadian Task Force on Preventive Health Care released guidelines for screening women at average breast cancer risk. Weak recommendations (framed using GRADE methodology) were made for screening women aged 50 to 74 years every two to three years, and for not screening women aged 40 to 49 years.

**Methods:** We conducted an interrupted time series analysis using administrative data to examine bilateral mammography use before and after a national guideline dissemination strategy targeting primary care physicians. Women aged 40 to 74 years living in Ontario or Alberta from 30 ^th^ November 2008 to 30 ^th^ November 2014 were included. Strata included age, region of residence, neighbourhood income quintile, immigration status, and education level.

**Results:** In both provinces, mammography use rates were lower in the post-intervention period (527 vs. 556 and 428 vs. 465/1000 participant-months - the monthly screening rate/1000 - in Ontario and Alberta, respectively). In Ontario, mammography trends decreased following guideline release to align with recommendations for women aged 40 to 74 (decrease of 2.21/1000 women, SE 0.26/1000, p<0.0001). In Alberta, mammography trends decreased for women aged 40 to 49 years (3/1000 women, SE 0.32, p<0.001) and 50 to 69 (2.9/1000 women, SE 0.79, p<0.001), but did not change for women aged 70 to 74 (0.7/1000 women, SE 1.23, p=0.553). In both provinces, trends in mammography use rates were sustained for up to three years after guideline release.

**Conclusions:** The guideline dissemination strategy appeared to increase uptake of guideline-concordant screening practice in women aged 40 to 49 years in Ontario and Alberta and for women aged 50 to 74 years in Ontario. Further work is required to understand these findings and whether shared decision making about mammography between women and providers increased among women considering mammography.

## Abbreviations

AKDN: Alberta Kidney Disease Network

CCI: Canadian Classification of Health Intervention procedure codes

CMAJ: Canadian Medical Association Journal

ICES: Institute for Clinical Evaluative Sciences

ITS: Interrupted Time Series

OBSP: Ontario Breast Screening Program

OCR: Ontario Cancer Registry

OHIP: Ontario Health Insurance Program

RPDB: Registered Persons Database

Task Force: Canadian Task Force on Preventive Health Care

## Introduction

The Canadian Task Force on Preventive Health Care (Task Force) was reconstituted in 2009 to develop and disseminate evidence-based clinical practice guidelines to support preventive practices in primary care. Breast cancer screening was the first topic selected for guideline development because the U.S. Preventive Services Task Force ^1^ had produced recommendations in 2009 favouring screening for women aged 40 years and older, yet questions about costs and benefits remained given the lower rate of breast cancer at younger ages and the potential risk of harms. ^2^

Breast cancer is the most common cancer in Canadian women with one in eight women receiving a diagnosis over her lifetime and one in thirty-three dying from the disease. In 2020, there were an estimated 27,400 new cases and 5,100 deaths from breast cancer, which is 13% of all cancer deaths in women in Canada. Mammography can identify asymptomatic breast cancer, but there are harms associated with screening, including overdiagnosis. ^3^ Several provinces and countries have implemented breast cancer screening programs with established targets; for instance, Alberta Health Services identified a target of screening 70% of eligible women aged 50 to 74 years every two years, which is consistent with the Canadian target of screening 70% of eligible women every 30 months.

In 2011, the Task Force aimed to provide a recent review of evidence to inform breast cancer screening practices. In keeping with the use of Grading of Recommendations Assessment, Development and Evaluation (GRADE) methodology, the Task Force reviewed available evidence using a systematic review to develop screening guidelines for women at average risk of breast cancer (see Box 1 for recommendations). ^4^ The Task Force developed weak recommendations based on moderate to low quality evidence. In keeping with GRADE methods, weak recommendations are those for which the benefits probably outweigh the harms, or the harms probably outweigh the benefits, but uncertainty exists. ^4, 5^ The guidelines also highlighted the need for clinicians to help patients make a decision that is consistent with the patient’s informed values and preferences. ^4^ This guideline was an update to the 2001 guideline published by the Task Force which recommended screening with mammography every one to two years for women aged 50 to 69. ^6, 7^

Box 1. **Task Force 2011 Breast Cancer Guideline Recommendations. ^4^**

*Recommendations follow the Grading of Recommendations Assessment, Development and Evaluation (GRADE) nomenclature*

• **For women aged 40–49 years** we recommend **not routinely** screening with mammography. ( *Weak recommendation; moderate quality evidence*)
• **For women aged 50–69 years** we recommend routinely screening with mammography every two to three years. ( *Weak recommendation; moderate quality evidence*)
• **For women aged 70–74 years** we recommend routinely screening with mammography every two to three years. ( *Weak recommendation; low quality evidence*)

The Task Force developed a tailored guideline dissemination strategy in partnership with stakeholder groups, including primary care physicians and women in the target age groups. We evaluated the impact of this dissemination strategy by investigating mammography use in Ontario and Alberta before and after the strategy was implemented.

The objective of this study was to determine, using an interrupted time series, the impact of the 2011 Task Force breast cancer screening guidelines on rates of mammography screening by age group in Ontario and Alberta. We hypothesized that there would be a significant change in mammography screening rates over time to align with the Task Force breast cancer recommendations, and that effects would be sustained for up to three years post-guideline release.

## Methods

We performed an interrupted time series (ITS) analysis to examine mammography use in Ontario and Alberta 36 months before (30 ^th^ November 2008 to 30 ^th^ November 2011) and after (1 ^st^ December 2011 to 30 ^th^ November 2014) the breast cancer screening guidelines were released in Canada in November 2011.

### Ethics statement

This study was approved by the Unity Health Toronto Research Ethics Board (REB# 12-220). Participant consent was waived by the REB, given all data analyses were conducted by ICES and Alberta Health Services using aggregate, non-identifiable data.

### Setting and participants

We included women aged 40 to 74 years who lived in Ontario or Alberta between November 2008 and November 2014. In Ontario, the Registered Persons Database (RPDB) and the Ontario Cancer Registry (OCR) were used to identify eligible participants. The databases were accessed and linked by ICES scientists ^8^^,^^9^ and not by members of the study team. The OCR database is considered the most comprehensive cancer registry in the province; the Institute for Clinical Evaluative Sciences (ICES) has access to data from January 1964 to present. Via a validated administrative algorithm, we identified eligible participants in Ontario; we used International Classification of Diseases for Oncology (ICD-O-3) C50 topography codes to identify women with a diagnosis of breast cancer. The algorithm is a sequence of programming codes in SAS software to assemble the cohort of people who were alive (i.e. no death date or death date was after the index date), were eligible for health care (i.e. date of end of eligibility was after the index date), and had an Ontario postal code at the index date. There is no available link to the SAS codes. Women with a history of breast cancer and/or a bilateral mastectomy before each period were excluded from the analysis. Bilateral mastectomy was identified using the Canadian Classification of Health Intervention (CCI) procedure codes. In Alberta, the Alberta Kidney Disease Network (AKDN) database (incorporating data from Alberta Health), which includes >99% of adult Albertans, was used to identify participants. ^10^ MT had access to de-identified data in the AKDN database; specifically, no health care numbers or names were available. For Alberta data, a validated administrative algorithm was used to identify and exclude women with a history of breast cancer using ICD-9 174 and ICD-10 C50 codes and 1 hospitalization or 2 claims in 2 years. ^11, 12^ Procedure codes from claims data (CCP codes 97.12A, 97.12B, and 97.22A) were used to exclude women with previous bilateral mastectomies (extended data).

### Intervention

The intervention was the Task Force’s guideline dissemination strategy, which included theory- and evidence-based printed and online education materials and a mass media campaign. ^13– 18^ The content of the education materials was based on the guideline recommendations, discussions with stakeholders about barriers to implementing the recommendations, and barriers to guideline implementation. The mass media campaign focused on key messages for the target audiences as identified from discussions with the Task Force and its stakeholders. The detailed approach to developing and testing the education materials is available online. The guidelines were published in the Canadian Medical Association Journal (CMAJ) on 21 ^st^ November 2011 accompanied by a mass media launch. ^4^ The print CMAJ (including the guidelines) was mailed to over 60,000 primary care and specialty physicians nationally. The printed education materials provided in this mailing included a one-page decision algorithm for patients and clinicians, and a one-page ‘Frequently Asked Questions’ document for patients and clinicians. These education materials were also available on the Task Force’s website along with a patient decision aid infographic explaining the risks and benefits of mammograms, a video and script that role modelled shared decision making (the process for patients and physicians to collectively make a decision regarding treatment), a presentation on the methods used to create the guidelines, and the systematic review used to inform guideline development. ^19^

### Outcomes

Data were cleaned to ensure that participants were adult and female. The Ontario and Alberta datasets were analysed separately and were not linked. NW conducted and oversaw the analysis of the Alberta data and RN oversaw the analysis of the Ontario data. Use of bilateral mammogram was the primary outcome, identified from the Ontario Health Insurance Program (OHIP, fee code in ‘X185’ and ‘X178’) and Ontario Breast Screening Program (OBSP) databases in Ontario and the AKDN claims data (CCP codes X27C, X27D, X27E) ^10^ in Alberta. For both Ontario and Alberta data, a mammogram was considered a duplicate if repeated within seven days and was then removed from the data; the date of the first mammogram within each time period was used for analysis. Additional variables collected included age group, setting (rural versus non-rural), income quintile, immigrant status, visible minority status ^1^, and education status. Urban area was defined as having a population of at least 1,000 and a density of 400 or more people per square kilometre. Income quintile was used as a proxy for the women’s socioeconomic status and was based on their postal code (neighbourhood) using Statistics Canada census data. Immigrant status and visible minority status were defined as the percentage of immigrants and visible minorities in the postal code, respectively. Education was specified as the proportion of women with a bachelor’s degree as the highest level of educational attainment. Details on immigrant, minority, and education status were not available for Alberta participants.

### Statistical analysis

Data were summarized descriptively using mammography counts and rates per 1000 participant-months. Data from each province were aggregated monthly to calculate rates (number of monthly screening tests/1000 participant-months) and ITS was performed using segmented regression. The model was defined as:

$${\text{Rate}}_{t}=\beta_{0}+\beta_{1}\times {\text{time}}_{t}+\beta_{2}\times {\text{intervention}}_{t}+\beta_{3}\times \text{time}\;{\text{after intervention}}_{t}\;+e_{t}$$

where Rate _t_ is the rate of breast cancer screening at each month t (t = 1 to 72 months) and e _t_ is the random error at time t. β _0_ is the intercept or pre-intervention baseline level of the rate; β _1_ is the slope of the rate until the intervention is introduced (slope prior to the intervention); β _2_ is the change in rate level that occurs in the period immediately following the introduction of intervention; and β _3_ is the change in rate trend (i.e. difference between post-intervention and pre-intervention slopes). We used this approach to test the significance of β _2_ (an immediate intervention effect) and β _3_ (significant intervention effect over time to determine sustained impact). ^21^

Subgroup analyses were performed for age categories. In Ontario, subgroup analyses were also separately performed by rural or urban setting, neighbourhood income quintile (mean income per person equivalent in an area obtained from census data), education categories (population in a neighbourhood with a bachelor’s degree), immigration categories (population in a neighbourhood of immigrant status), and minority categories (created as a % of visible minority in a total population by postal code). The cut off values for categories were determined using distributions of the 1 ^st^ and 3 ^rd^ quartiles (interquartile range) of the neighbourhood distributions at pre- and post-intervention periods.

For Ontario data, regression parameters were estimated by maximum likelihood using SAS Enterprise Guide, version 7.15 software procedure AUTOREG (SAS Institute Inc., Cary, NC, USA), which adjusts for autocorrelation. ^22, 23^ For Alberta data, analysis was conducted using Stata MP 15.1 software command ‘itsa’; however, the same models and parameters were used. ^24^ Canadian mammography screening is recommended every 24 to 36 months ^25^; final models included autogressive terms for only up to 12 lags, selected by backward elimination. The Durbin-Watson statistic was calculated to test autocorrelation terms. All statistical tests were two-sided and statistical significance was defined as a p-value less than 0.05.

## Results

### Ontario

*Descriptive*

A total of 2,935,241 women were included before guideline dissemination and 3,098,205 women were included after dissemination; the cohorts had similar demographic characteristics ( Table 1). Overall, the number of women who had a mammogram decreased in the post-intervention period compared to pre-intervention (527 versus 556 per 1000 participant-months, respectively). Similar trends were observed in women aged 40 to 49 and 50 to 69 years. The proportion of women aged 70 to 74 years who underwent a mammogram was slightly higher in the post-intervention period ( Table 1). Both at pre- and post-guideline release, women in higher income quintiles (compared to lower quintiles) and women in rural areas (compared to urban areas) had higher screening mammography use.

**Table 1.** Participant characteristics (extended data).

	Pre-guideline		Post-guideline	
Characteristics	Total (N)	Screened with mammogram N (per 1000)	Total (N)	Screened with mammogram N (per 1000)
**Ontario data**				
Overall	2,935,241	1,630,535 (556)	3,098,205	1,633,248 (527)
Age group				
40-49	1,050,145	339,728 (324)	1,018,506	250,974 (246)
50-69	1,657,828	1,141,380 (688)	1,825,012	1,213,113 (665)
70-74	227,268	149,427 (657)	254,687	169,161 (664)
Income quintile			3,098,205	
1	516,929	249,585 (483)	591,497	275,248 (465)
2	564,645	302,604 (536)	612,049	314,777 (514)
3	584,511	326,112 (558)	614,834	328,724 (535)
4	626,764	363,790 (580)	610,857	334,676 (548)
5	632,732	383,991 (607)	665,303	378,271 (569)
Place of residence				
Urban	2,579,041	1,430,293 (555)	2,754,946	1,447,086 (525)
Rural	356,175	200,233 (562)	339,661	184,638 (544)
Immigration status				
Low	649,391	364,866 (562)	676,066	367,600 (544)
Moderate	1,302,240	742,379 (570)	1,352,420	729,345 (539)
High	650,969	344,527 (529)	676,104	338,386 (500)
Education level				
Low	651,732	354,736 (544)	680,395	358,288 (527)
Moderate	1,307,640	729,642 (558)	1,352,696	720,646 (533)
High	650,806	370,766 (570)	679,739	360,033 (530)
**Alberta data**				
Overall	788,131	366,547 (465)	861,014	368,735 (428)
Age group				
40-49	291,601	102,225 (351)	298,891	82,822 (277)
50-69	445,294	239,115 (537)	503,230	257,375 (511)
70-74	51,236	25,207 (492)	58,893	28,538 (485)
Income quintile				
1	129,739	55,125 (425)	138,547	54,365 (392)
2	142,411	67,369 (473)	150,603	65,165 (433)
3	141,065	72,418 (513)	150,079	70,471 (470)
4	149,755	79,936 (534)	162,997	79,362 (487)
5	156,731	88,649 (566)	168,915	87,162 (516)
Place of residence				
Urban	645,739	335,744 (520)	713,032	339,172 (476)
Rural	77,577	29,662 (382)	80,320	28,407 (354)

*ITS results*

**Figure 1.**
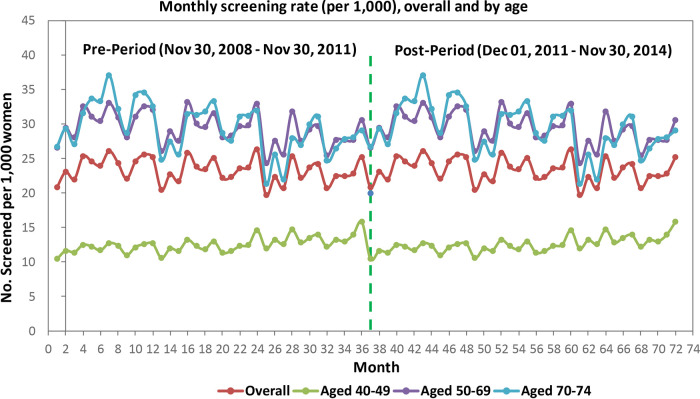
Ontario data.

Among women aged 40 to 49 years, monthly screening mammography rates increased before the guideline release (slope 0.06/1000, SE 0.01/1000, p < 0.0001). Immediately following the guideline release, there was a significant rate decrease of 2.21 per 1000 women (SE 0.26/1000, p < 0.0001) who received a mammogram. This rate was sustained; however, the slope was not, meaning the rate of women who received a mammogram did not continue to decrease further during this period ( Table 2, Figure 1).

**Table 2.** Interrupted time series results for Ontario data (per 1000).

Outcome	Regression coefficients	Estimate	Std Err	Probt
All women	Pre-intervention baseline rate level	24.2589	0.5508	0.0000
	Pre-intervention slope	−0.0551	0.0144	0.0003
	Change in rate level (immediate effect post-intervention)	1.9574	0.2978	0.0000
	Change in rate trend (sustained impact post-intervention)	0.0034	0.0210	0.8703
Women aged 40-49	Pre-intervention baseline rate level	11.5055	0.2881	0.0000
	Pre-intervention slope	0.0619	0.0111	0.0000
	Change in rate level (immediate effect post-intervention)	−2.2084	0.2570	0.0000
	Change in rate trend (sustained impact post-intervention)	<0.0001	0.0168	0.9985
Women aged 50-69	Pre-intervention baseline rate level	31.1162	0.7051	0.0000
	Pre-intervention slope	−0.1084	0.0187	0.0000
	Change in rate level (immediate effect post-intervention)	3.8229	0.3880	0.0000
	Change in rate trend (sustained impact post-intervention)	0.0072	0.0273	0.7941
Women aged 70-74	Pre-intervention baseline rate level	32.9099	1.0495	0.0000
	Pre-intervention slope	−0.2084	0.0207	0.0000
	Change in rate level (immediate effect post-intervention)	7.4953	0.4141	0.0000
	Change in rate trend (sustained impact post-intervention)	0.0028	0.0295	0.9236
Income Q1	Pre-intervention baseline rate level	20.1457	0.5069	0.0000
	Pre-intervention slope	−0.0323	0.0130	0.0158
	Change in rate level (immediate effect post-intervention)	1.1814	0.2688	0.0000
	Change in rate trend (sustained impact post-intervention)	0.0006	0.0190	0.9754
Income Q2	Pre-intervention baseline rate level	22.9990	0.5525	0.0000
	Pre-intervention slope	−0.0459	0.0155	0.0042
	Change in rate level (immediate effect post-intervention)	1.7047	0.3240	0.0000
	Change in rate trend (sustained impact post-intervention)	−0.0007	0.0227	0.9763
Income Q3	Pre-intervention baseline rate level	24.0921	0.5490	0.0000
	Pre-intervention slope	−0.0472	0.0147	0.0021
	Change in rate level (immediate effect post-intervention)	1.6950	0.3058	0.0000
	Change in rate trend (sustained impact post-intervention)	0.0023	0.0215	0.9155
Income Q4	Pre-intervention baseline rate level	25.6099	0.5823	0.0000
	Pre-intervention slope	−0.0629	0.0158	0.0002
	Change in rate level (immediate effect post-intervention)	2.2163	0.3281	0.0000
	Change in rate trend (sustained impact post-intervention)	0.0049	0.0231	0.8317
Income Q5	Pre-intervention baseline rate level	27.6219	0.5892	0.0000
	Pre-intervention slope	−0.0806	0.0157	0.0000
	Change in rate level (immediate effect post-intervention)	2.7806	0.3246	0.0000
	Change in rate trend (sustained impact post-intervention)	0.0090	0.0229	0.6952
Education 1	Pre-intervention baseline rate level	23.3601	0.5941	0.0000
	Pre-intervention slope	−0.0486	0.0156	0.0026
	Change in rate level (immediate effect post-intervention)	1.7650	0.3218	0.0000
	Change in rate trend (sustained impact post-intervention)	0.0012	0.0227	0.9577
Education 2	Pre-intervention baseline rate level	24.3047	0.5652	0.0000
	Pre-intervention slope	−0.0568	0.0142	0.0002
	Change in rate level (immediate effect post-intervention)	2.0191	0.2924	0.0000
	Change in rate trend (sustained impact post-intervention)	0.0032	0.0207	0.8783
Education 3	Pre-intervention baseline rate level	25.7350	0.5303	0.0000
	Pre-intervention slope	−0.0706	0.0159	0.0000
	Change in rate level (immediate effect post-intervention)	2.4675	0.3360	0.0000
	Change in rate trend (sustained impact post-intervention)	0.0067	0.0234	0.7770
Immigration 1	Pre-intervention baseline rate level	24.2314	0.6186	0.0000
	Pre-intervention slope	−0.0486	0.0171	0.0059
	Change in rate level (immediate effect post-intervention)	1.7023	0.3565	0.0000
	Change in rate trend (sustained impact post-intervention)	0.0050	0.0250	0.8414
Immigration 2	Pre-intervention baseline rate level	25.2624	0.5641	0.0000
	Pre-intervention slope	−0.0705	0.0147	0.0000
	Change in rate level (immediate effect post-intervention)	2.4623	0.3029	0.0000
	Change in rate trend (sustained impact post-intervention)	0.0060	0.0214	0.7784
Immigration 3	Pre-intervention baseline rate level	23.0136	0.5425	0.0000
	Pre-intervention slope	−0.0431	0.0148	0.0049
	Change in rate level (immediate effect post-intervention)	1.6446	0.3082	0.0000
	Change in rate trend (sustained impact post-intervention)	−0.0029	0.0217	0.8956
Minority 1	Pre-intervention baseline rate level	24.8682	0.6375	0.0000
	Pre-intervention slope	−0.0547	0.0168	0.0017
	Change in rate level (immediate effect post-intervention)	1.9096	0.3467	0.0000
	Change in rate trend (sustained impact post-intervention)	0.0054	0.0244	0.8253
Minority 2	Pre-intervention baseline rate level	25.1783	0.5621	0.0000
	Pre-intervention slope	−0.0695	0.0149	0.0000
	Change in rate level (immediate effect post-intervention)	2.4343	0.3074	0.0000
	Change in rate trend (sustained impact post-intervention)	0.0054	0.0217	0.8043
Minority 3	Pre-intervention baseline rate level	22.4733	0.5244	0.0000
	Pre-intervention slope	−0.0390	0.0145	0.0090
	Change in rate level (immediate effect post-intervention)	1.4664	0.3030	0.0000
	Change in rate trend (sustained impact post-intervention)	−0.0016	0.0213	0.9413
Urban	Pre-intervention baseline rate level	24.2510	0.5299	0.0000
	Pre-intervention slope	−0.0548	0.0143	0.0003
	Change in rate level (immediate effect post-intervention)	1.9529	0.2963	0.0000
	Change in rate trend (sustained impact post-intervention)	0.0030	0.0209	0.8849
Rural	Pre-intervention baseline rate level	24.3173	0.7912	0.0000
	Pre-intervention slope	−0.0569	0.0186	0.0031
	Change in rate level (immediate effect post-intervention)	1.9840	0.3781	0.0000
	Change in rate trend (sustained impact post-intervention)	0.0062	0.0268	0.8169

Among women aged 50 to 69 years, monthly screening mammography rates decreased before the guideline release ( Table 2). Immediately following the guideline release, there was a significant increase of 3.82 per 1000 women who received a mammogram. This rate was sustained; however, the slope was not.

Among women aged 70 to 74 years, monthly screening mammography rates decreased before the guideline release ( Table 2). Immediately following the guideline release, there was a significant increase of 7.50 per 1000 women who received a mammogram. This rate was sustained three years following the intervention.

Among all women, rates of mammography screening were significantly declining pre-guideline release; following the release, a significant decrease in mammography use was observed in all income quintiles and across all education levels. There were no significant changes to the slopes in the three years following the guideline release. Similar trends (significantly decreasing slope, increase in mammography use, no significant difference in slope three years after guideline release) were observed across neighbourhoods with a low, moderate, or high percentage of women who were immigrants or identified as a visible minority and among women in urban and rural areas ( Table 2).

### Alberta

*Descriptive*

A total of 788,131 women were included pre-intervention and 861,014 women were included post-intervention; demographic details are provided in Table 1. Overall, mammography use decreased post-intervention compared to pre-intervention (428 versus 465 per 1000 participant-months, respectively).

These trends were observed in women aged 40 to 74 years. In both the pre- and post-intervention periods, women in higher income quintiles had increased rates of mammography use compared to women in low income quintiles ( Table 1). In Alberta, women residing in urban areas had higher rates of mammography use pre- and post-intervention as compared to women residing in rural areas.

*ITS results*

Among women aged 40 to 49 years, monthly screening mammography rates in Alberta were slightly decreasing before guideline release. Immediately following guideline release, there was a significant rate decrease of 3 per 1000 women who received a mammogram. This rate was sustained; however, the rate of women who received a mammogram did not decrease further during this period (post- minus pre-intervention slope difference 0.0015/1000, SE 0.02/1000, p = 0.933).

**Figure 2.**
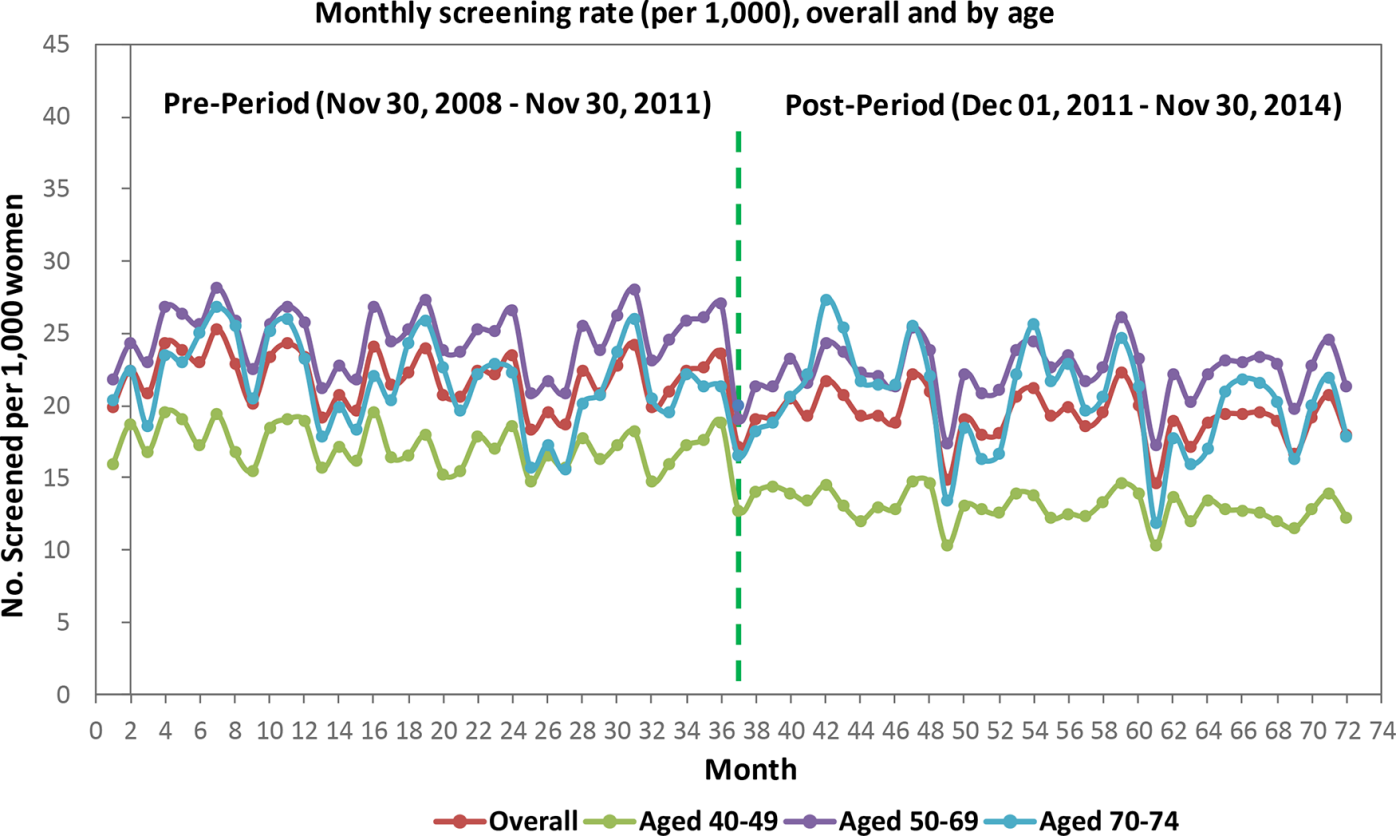
Alberta data.

Among women aged 50 to 69 years, monthly screening mammography rates were stable. Immediately following the guideline release, there was a significant decrease of 2.9 per 1000 women who received a mammogram; this rate was sustained for the remainder of the study period ( Figure 2, Table 3).

**Table 3.** Interrupted time series results for Alberta data (per 1000).

Outcome	Regression coefficients	Estimate	StdErr	Probt
All women	Pre-intervention baseline rate level	22.4458	0.4425	<0.001
	Pre-intervention slope	−0.0253	0.0230	0.275
	Change in rate level (immediate effect post-intervention)	−1.8754	0.6446	0.005
	Change in rate trend (sustained impact post-intervention)	0.00002	0.0228	0.999
Women aged 40-49	Pre-intervention baseline rate level	17.8318	0.2892	<0.001
	Pre-intervention slope	−0.0330	0.0151	0.033
	Change in rate level (immediate effect post-intervention)	−3.0380	0.3222	<0.001
	Change in rate trend (sustained impact post-intervention)	0.0015	0.0181	0.933
Women aged 50-69	Pre-intervention baseline rate level	24.5529	0.5153	<0.001
	Pre-intervention slope	0.0104	0.0271	0.701
	Change in rate level (immediate effect post-intervention)	−2.9100	0.7874	<0.001
	Change in rate trend (sustained impact post-intervention)	0.0031	0.0262	0.906
Women aged 70-74	Pre-intervention baseline rate level	22.9821	0.6908	<0.001
	Pre-intervention slope	−0.0671	0.0335	0.050
	Change in rate level (immediate effect post-intervention)	0.7317	1.2270	0.553
	Change in rate trend (sustained impact post-intervention)	0.0095	0.0426	0.825
Income Q1	Pre-intervention baseline rate level	18.8552	0.4006	<0.001
	Pre-intervention slope	0.0178	0.0188	0.346
	Change in rate level (immediate effect post-intervention)	−2.3166	0.4793	<0.001
	Change in rate trend (sustained impact post-intervention)	−0.0244	0.0208	0.246
Income Q2	Pre-intervention baseline rate level	21.9090	0.4367	<0.001
	Pre-intervention slope	−0.0076	0.0230	0.743
	Change in rate level (immediate effect post-intervention)	−2.2720	0.6255	0.001
	Change in rate trend (sustained impact post-intervention)	−0.0132	0.0237	0.580
Income Q3	Pre-intervention baseline rate level	24.4766	0.5242	<0.001
	Pre-intervention slope	−0.0208	0.0269	0.443
	Change in rate level (immediate effect post-intervention)	−2.4265	0.7449	0.002
	Change in rate trend (sustained impact post-intervention)	−0.0036	0.0262	0.891
Income Q4	Pre-intervention baseline rate level	26.1657	0.5731	<0.001
	Pre-intervention slope	−0.0346	0.0294	0.243
	Change in rate level (immediate effect post-intervention)	−2.2011	0.7877	0.007
	Change in rate trend (sustained impact post-intervention)	−0.0048	0.0289	0.867
Income Q5	Pre-intervention baseline rate level	28.7066	0.5406	<0.001
	Pre-intervention slope	−0.0621	0.0270	0.025
	Change in rate level (immediate effect post-intervention)	−2.1283	0.8175	0.011
	Change in rate trend (sustained impact post-intervention)	0.0219	0.0265	0.411
Urban	Pre-intervention baseline rate level	25.1714	0.4877	<0.001
	Pre-intervention slope	−0.0313	0.0255	0.23
	Change in rate level (immediate effect post-intervention)	−2.1717	0.7107	0.003
	Change in rate trend (sustained impact post-intervention)	0.0040	0.0253	0.876
Rural	Pre-intervention baseline rate level	16.8876	0.3954	<0.001
	Pre-intervention slope	0.0310	0.0185	0.098
	Change in rate level (immediate effect post-intervention)	−2.4010	0.5681	<0.001
	Change in rate trend (sustained impact post-intervention)	−0.0589	0.0214	0.008

Among women aged 70 to 74 years, monthly screening mammography rates were stable before the guideline release. Immediately following the guideline release, there were no significant changes in the rate of women aged 70 to 74 who received a mammogram.

All strata (by age, income quintile, rural/urban region) experienced significant drops in mammography rates at intervention, with the exception of women 70 to 74 years. Prior to the intervention, most slopes were stable pre- and post-intervention with the exception of women 40 to 49 years (rate decreased immediately post-intervention) and women in high income quintiles, who demonstrated a slight decrease in mammography use prior to guideline release (Income Q5, see Table 3). Trends were stable three years following guideline release, with the exception of women living in rural areas, for whom mammography rates continued to decrease over time.

## Discussion

In comparing rates of mammography screening following guideline dissemination, we observed trend differences in the two provinces. Overall, Alberta women underwent less mammography screening both pre- and post-guideline dissemination compared to Ontario women. In both provinces, fewer women underwent mammography in the 36 months after guideline dissemination compared to the same period before guidelines were released. In Ontario, mammography rates among women aged 40 to 49 years decreased immediately following guideline release and increased for women aged 50 to 74, in keeping with the guideline recommendations. In Alberta, mammography use decreased for women aged 40 to 69 and did not significantly change among women aged 70 to 74. In Ontario, mammography use decreased overall following guideline release across all income quintiles and education levels and among women living in both urban and rural areas. Similarly, in Alberta, mammography decreased among women in all income quintiles and decreased for women living in both urban and rural areas; the rate of mammography use continued to decrease among women living in rural areas in Alberta.

In both Ontario and Alberta, mammography among women aged 40 to 49 years immediately decreased following guideline release. This decrease may correlate with the difference in screening recommendations in the 2011 guideline compared to the previous 2001 Task Force guideline, which suggested that upon reaching the age of 40, women should consider the benefits and risks of screening to determine whether to begin routine mammography between 40 and 49 or whether to delay until age 50. ^7^ Similarly, rates of mammography among women aged 50 to 59 in Alberta may have decreased following the recommendation for extending screening intervals to 2 to 3 versus 1 to 2 years. ^25, 26^

Our results are similar to findings reported in the United States (U.S.), where an overall decline in mammography for women aged 40 to 49 years was observed post-dissemination of the 2009 U.S. Preventive Services Task Force guidelines. ^27– 29^ For women aged 50 to 69 years, U.S. studies showed a decline in mammography following release of their national guidelines, which were similar to the Task Force recommendations. ^27– 29^ Given the Canadian Task Force’s weak (or ‘conditional’) recommendation to screen women aged 50 to 69 and advice to engage in shared decision making about screening, fewer women may have decided not to undergo screening. ^4^

Higher income levels were associated with more screening. These results are similar to those from other studies of breast cancer screening conducted in the U.S. ^30– 34^ and Canada. ^35^ In Ontario, women living in rural areas had higher mammogram screening rates compared to women living in urban areas; this may indicate the effect of organized screening programs in rural communities. Additional studies further examining trends in Canadian breast cancer screening, particularly following the most recent 2018 release of the Task Force's breast cancer guideline update, may provide further insights into these trends.

There are several limitations to this study. Firstly, individual level data (such as exact age, education status, ethnicity) were not available; rather, these data were available as categories, and education and immigration status were only available at a neighbourhood level. Consequently, we were not able to investigate the impact of patient level factors on screening activities. Secondly, there may have been other interventions targeting breast cancer screening in Ontario and Alberta during the study periods. In Ontario, for instance, Cancer Care Ontario launched a campaign to increase screening for breast, cervical, and prostate cancer in November 2012. This media campaign encouraged people to send cards to friends and family to undergo cancer screening. However, the Task Force engaged provincial partners on the guidelines and we were not able to identify any large-scale strategies that may have overlapped with dissemination. An additional confounder may be that pay-per-performance models (meaning practitioners receive compensation based on their activities, such as screening, and not a standard salary) were introduced in Ontario at this time; however, the models don’t definitively explain the trends observed in this study, as the literature is not conclusive on the impact of these models on rates of breast cancer screening. ^36, 37^ In addition, we were not able to assess the use of shared decision making, which was recommended in the guideline, as these data are not captured in administrative databases. Finally, these data were not created to answer the specific research question, which is a limitation of all population-level administrative database studies.

In summary, the decline in mammography for women aged 40 to 49 years was in alignment with guideline recommendations. Future strategies should focus on optimizing implementation of the recommendations for women aged 50 to 74 years. This requires understanding barriers to behavioural changes at the clinician and patient level and investigating the use of shared decision making.

## Data availability

### Underlying data

The datasets generated and analysed during the current study are not publicly available. They could potentially be made available with assistance from the corresponding author, if for the purpose of further research. Obtaining Ontario data which are owned by the Ontario Ministry of Health and Long-Term Care would require providing a research protocol and consultation with ICES, who will also request a budget be submitted for data access and analysis. Researchers could alternatively request to obtain a similar dataset for Alberta or Ontario data.

### Extended data

Open Science Framework: Changes in mammography screening in Ontario and Alberta following national guideline dissemination: an interrupted time series analysis. http://doi.org/10.17605/OSF.IO/5H2T8. ^38^

This project contains the following extended data:

- Supplementary File 1: Sustaining Change_OntarioandAlbertaPopulationData
- Supplementary File 2: Supplementary File 2_Sustaining Change_OntarionandAlbertaCodes

Data are available under the terms of the CC0 1.0 Universal (CC0 1.0) Public Domain Dedication.

## Disclaimer

This study is based in part by data provided by Alberta Health and Alberta Health Services. The interpretation and conclusions contained herein are those of the researchers and do not represent the views of the Government of Alberta or Alberta Health Services. Neither the Government of Alberta nor Alberta Health Services express any opinion in relation to this study.

## Footnotes

^1^
‘Visible minority’ is a term used in the ICES database. ^20^
