# Supplementary material for: Changes in mammography screening in Ontario and Alberta following national guideline dissemination: an interrupted time series analysis
Source: F1000Res. 2022 Nov 7;10:1044. Originally published 2021 Oct 15. [Version 2] doi: 10.12688/f1000research.55004.2 (PMC9745205; doi:10.12688/f1000research.55004.2)
Supplement: Peer review history

# Peer review: Reviewer response for version 2

**DOI:** 10.5256/f1000research.140684.r155166

**Published:** 2022-12-12

**Authors:** Jacqueline M. Hirth, Judith Gutierrez, Tiffany Ostovar-Kermani

**Affiliations:** 1 Baylor College of Medicine, Houston, USA

The authors have addressed all comments. While we feel that the dissemination strategy is not an intervention that should be included separately from the change in guidelines, as they were not assessed individually, we accept the changes as they are.

Is the work clearly and accurately presented and does it cite the current literature?

Yes

If applicable, is the statistical analysis and its interpretation appropriate?

Yes

Are all the source data underlying the results available to ensure full reproducibility?

Partly

Is the study design appropriate and is the work technically sound?

Partly

Are the conclusions drawn adequately supported by the results?

Partly

Are sufficient details of methods and analysis provided to allow replication by others?

Yes

Reviewer Expertise:

Population health, primary and secondary cancer prevention, women's health

We confirm that we have read this submission and believe that we have an appropriate level of expertise to confirm that it is of an acceptable scientific standard.

# Peer review: Reviewer response for version 2

**DOI:** 10.5256/f1000research.140684.r155167

**Published:** 2022-11-08

**Authors:** Sisse Helle Njor

**Affiliations:** 1 Randers Regional Hospital, Randers, Denmark; 2 Aarhus University, Aarhus, Denmark

No further comments.

Is the work clearly and accurately presented and does it cite the current literature?

Yes

If applicable, is the statistical analysis and its interpretation appropriate?

Yes

Are all the source data underlying the results available to ensure full reproducibility?

Yes

Is the study design appropriate and is the work technically sound?

Partly

Are the conclusions drawn adequately supported by the results?

Partly

Are sufficient details of methods and analysis provided to allow replication by others?

Partly

Reviewer Expertise:

Evaluation of cancer screening

I confirm that I have read this submission and believe that I have an appropriate level of expertise to confirm that it is of an acceptable scientific standard.

# Peer review: Reviewer response for version 1

**DOI:** 10.5256/f1000research.58537.r148598

**Published:** 2022-09-20

**Authors:** Jacqueline M. Hirth, Judith Gutierrez, Tiffany Ostovar-Kermani

**Affiliations:** 1 Baylor College of Medicine, Houston, USA

This manuscript described the change in mammography screening, as a rate of screenings per 1000 participant months, in Ontario and Alberta, Canada, before and after a national change in guideline on mammography. While the topic is important in assessing the impact of guidelines on practice, there are some issues the authors may want to consider addressing to strengthen their manuscript.

 General

1. In the abstract and body of the paper, the new guidelines are described as “weak recommendations.” This description gives the impression that the authors may feel that the guidelines are inappropriate. Therefore, we suggest that this wording be amended to, “recommendations based on weak evidence.” This would help an international audience get a clearer picture of what is meant by the authors.
2. The purpose of the paper is not well-developed. There is mention of a dissemination strategy, which is discussed in the methods, but does not seem to be tested for or mentioned in the conclusion. If there was no comparison for differences in dissemination strategies, then it appears that the manuscript evaluates the effect of the guideline change on mammography rates, but not dissemination strategy of the guidelines. We recommend changing the second to last paragraph in the introduction to reflect that dissemination strategy was not assessed and the methods section to reflect that it was the change in guidelines that was assessed rather than the dissemination strategy.

Abstract

1. Amend description of “weak recommendations” to “recommendations based on weak evidence.”
2. Recommend removing information about dissemination strategy in line with recommendations above.
3. Recommend clarifying that “trends in mammography use rates” be changed to “declining trends in mammography use rates” in last sentence of results in abstract. Consider removing standard errors to stay within word limit.
4. Recommend changing conclusion to indicate that it was the guideline that changed uptake, rather than the dissemination strategy.
5. The conclusion does not match the results in the abstract. The results indicate a decrease in mammography trends, but the conclusion indicates that there was an increased uptake in guideline-concordant screening practice. As this was not actually assessed (would require individual level data) the conclusion would be more accurate if discussed along the lines of, “decreasing trends in mammography rates among 40-49 year old women indicated that this group may be receiving fewer screenings, possibly in response to the new guidelines.”

Introduction

1. The dissemination strategy was not elaborated on in the introduction. Since this study did not test the dissemination strategy, it is recommended that the description of the strategy be moved from Methods section to the Introduction section.
2. Change “weak recommendations” to “recommendations based on weak evidence.”

Methods

1. We recommend reorganization of the methods section.
  1. Begin with the setting and participants with integration of the dates of data collection in the current first sentence, followed by the ethics statement then Outcomes and statistical analysis.
  2. We recommend that the description of the dissemination strategy be shortened and included in the Introduction section.
  3. We recommend that the statistical analyses used (ITS) be moved to the statistical analyses section. The method used for this study looked like a retrospective population surveillance study.
  4. Provide a justification for using interrupted times series analyses in the statistical analyses section.

Results

1. Were the women included in the study cohorts? This was mentioned in sentence 1 of Results section. With shifting ages across the timeline, it seems they could be described as the “study population,” as women would shift into different age groups across the times assessed.
2. Recommend using p-value designation of <0.001 in last column of tables where currently 0.0000 is listed, as these are more accepted statistical notations in the literature.
3. In the Alberta results section, Alberta was mentioned in the text as well as subheading. Would be more clear if the Ontario section was written in similar fashion. At first I thought that the total population estimates were for the entire study, not just Ontario.

Discussion

1. It appears new results are added in first paragraph. We saw no evidence of direct comparisons of mammography rates between Alberta and Ontario in the results section. Recommended to amend statistical analyses section and results section accordingly. The first paragraph of the Discussion section could then be the final paragraph of the Results section.
2. Last sentence in 3 ^rd^ paragraph not clear. If fewer women decided not to undergo screening, wouldn’t the trend have been positive after guideline implementation?
3. Consider subheadings for Limitations and Conclusion sections.
4. The Conclusion section in the Discussion appears to differ from the conclusion in the Abstract.
5. Given the importance placed on shared decision-making in the conclusion, more context could be given in the Introduction and/or Discussion section(s).

Is the work clearly and accurately presented and does it cite the current literature?

Yes

If applicable, is the statistical analysis and its interpretation appropriate?

Yes

Are all the source data underlying the results available to ensure full reproducibility?

Partly

Is the study design appropriate and is the work technically sound?

Partly

Are the conclusions drawn adequately supported by the results?

Partly

Are sufficient details of methods and analysis provided to allow replication by others?

Yes

Reviewer Expertise:

Population health, primary and secondary cancer prevention, women's health

We confirm that we have read this submission and believe that we have an appropriate level of expertise to confirm that it is of an acceptable scientific standard, however we have significant reservations, as outlined above.

# Response

**Published:** 2022-11-02

**Authors:** Christine Fahim

**Affiliations:** St. Michael's Hospital, Toronto, Canada

Response to Reviewer 2

 This manuscript described the change in mammography screening, as a rate of screenings per 1000 participant months, in Ontario and Alberta, Canada, before and after a national change in guideline on mammography. While the topic is important in assessing the impact of guidelines on practice, there are some issues the authors may want to consider addressing to strengthen their manuscript.

 General

 1. In the abstract and body of the paper, the new guidelines are described as “weak recommendations.” This description gives the impression that the authors may feel that the guidelines are inappropriate. Therefore, we suggest that this wording be amended to, “recommendations based on weak evidence.” This would help an international audience get a clearer picture of what is meant by the authors.

*Thank you for this comment. Our language is consistent with that of GRADE methodology (https://bestpractice.bmj.com/info/toolkit/learn-ebm/what-is-grade/; PMCID: PMC4364259) that provides a tool for rating the quality of evidence for clinical guidelines. GRADE recommendations can be ‘strong’ or ‘weak’ depending on the strength of the evidence. According to the GRADE definition, “weak recommendations imply that there is likely to be an important variation in the decision that informed persons are likely to make.. a weak recommendation indicates that engaging in a shared decision making process is essential, while a strong recommendation suggests that it is not usually necessary to present both options” (Siemieniuk and Guyatt, BMJ). Because the Canadian Task Force used GRADE methodology to develop their guideline and recommendations, we believe it is important to refer to the terms that the guideline developers used. However, we appreciate the author’s guidance that ‘weak recommendations’ may be unclear and we have ensured details around GRADE methodology have been included in the introduction.*

 2. The purpose of the paper is not well-developed. There is mention of a dissemination strategy, which is discussed in the methods, but does not seem to be tested for or mentioned in the conclusion. If there was no comparison for differences in dissemination strategies, then it appears that the manuscript evaluates the effect of the guideline change on mammography rates, but not dissemination strategy of the guidelines. We recommend changing the second to last paragraph in the introduction to reflect that dissemination strategy was not assessed and the methods section to reflect that it was the change in guidelines that was assessed rather than the dissemination strategy.

*Thank you for this important point. The dissemination strategy was passive and was disseminated via avenues considered ‘routine’ for primary care practitioners (including via a leading Canadian medical journal, at conferences, etc.) The aim was to determine mammography trends following release of the guideline and the dissemination strategy, rather than test the dissemination strategy in a controlled study design. We recognize that the findings may not be causally linked to the dissemination strategy, and we have amended our introduction to clarify the purpose and outline potential confounders in our limitations section. We have also clarified the study objective in the introduction.*

 Abstract

 1. Amend description of “weak recommendations” to “recommendations based on weak evidence.”

*In order to remain consistent with GRADE language, we have revised this sentence to “weak recommendations based on GRADE terminology (i.e., the benefits and risks are closely balanced and/or uncertain).*

 2. Recommend removing information about dissemination strategy in line with recommendations above.

*Thank you, we have removed “dissemination strategy targeting primary care physicians” from the abstract and the sentence in the abstract now reads “We conducted an interrupted time series analysis using administrative data to examine bilateral mammography use before and after release of a national breast cancer screening guideline”.*

 3. Recommend clarifying that “trends in mammography use rates” be changed to “declining trends in mammography use rates” in last sentence of results in abstract. Consider removing standard errors to stay within word limit.

*Thank you for this comment. The trends did not decline in all cases. While we saw a decrease in screening for all women in Ontario, and for women aged 40-69 in Alberta, we did not see a decrease among women aged 70-74 in Alberta.*

 4. Recommend changing conclusion to indicate that it was the guideline that changed uptake, rather than the dissemination strategy.

*Thank you, we have revised the concluding sentence.*

   

 5. The conclusion does not match the results in the abstract. The results indicate a decrease in mammography trends, but the conclusion indicates that there was an increased uptake in guideline-concordant screening practice. As this was not actually assessed (would require individual level data) the conclusion would be more accurate if discussed along the lines of, “decreasing trends in mammography rates among 40-49 year old women indicated that this group may be receiving fewer screenings, possibly in response to the new guidelines.”

*Thank you for this comment. We have revised the wording as suggested by the reviewer.*

 Introduction

 1. The dissemination strategy was not elaborated on in the introduction. Since this study did not test the dissemination strategy, it is recommended that the description of the strategy be moved from Methods section to the Introduction section.

*Thank you; our goal was to evaluate the impact of guideline release, which was disseminated using a national strategy. We thus have kept the description of the strategy in the methods section. We recognize there are limitations to drawing conclusions of impact of the strategy, which we have outlined in the limitations.*

 2. Change “weak recommendations” to “recommendations based on weak evidence.”

*Please see comments above regarding consistency with GRADE language.*

 Methods

 We recommend reorganization of the methods section.

 1. Begin with the setting and participants with integration of the dates of data collection in the current first sentence, followed by the ethics statement then Outcomes and statistical analysis.

*Thank you for this suggestion. We have reorganized the methods section accordingly.*

 2. We recommend that the description of the dissemination strategy be shortened and included in the Introduction section.

*Thank you, please see above comment for our rationale.*

 3. We recommend that the statistical analyses used (ITS) be moved to the statistical analyses section. The method used for this study looked like a retrospective population surveillance study.

*Thank you, we have provided details on the ITS in the statistical analysis section.*

 4. Provide a justification for using interrupted times series analyses in the statistical analyses section.

*Thank you, this has been provided.*

 Results

 1. Were the women included in the study cohorts? This was mentioned in sentence 1 of Results section. With shifting ages across the timeline, it seems they could be described as the “study population,” as women would shift into different age groups across the times assessed.

*Thank you for this comment. We have revised “cohorts” to “study populations”.*

 2. Recommend using p-value designation of <0.001 in last column of tables where currently 0.0000 is listed, as these are more accepted statistical notations in the literature.

*Thank you for this suggestion, we have updated the tables accordingly.*

 3. In the Alberta results section, Alberta was mentioned in the text as well as subheading. Would be more clear if the Ontario section was written in similar fashion. At first I thought that the total population estimates were for the entire study, not just Ontario.

*Thank you for this suggestion, we have added “in Ontario” to provide clarity for this section.*

 Discussion

 1. It appears new results are added in first paragraph. We saw no evidence of direct comparisons of mammography rates between Alberta and Ontario in the results section. Recommended to amend statistical analyses section and results section accordingly. The first paragraph of the Discussion section could then be the final paragraph of the Results section.

*Thank you. We have moved the first paragraph of the discussion to the results.*

 2. Last sentence in 3rd paragraph not clear. If fewer women decided not to undergo screening, wouldn’t the trend have been positive after guideline implementation?

*We observed a decrease in screening rates for all women, except for those aged 70-74 in Alberta.*

 3. Consider subheadings for Limitations and Conclusion sections.

*Thank you for this suggestion. We have added subheadings for Limitations and Conclusions.*

 4. The Conclusion section in the Discussion appears to differ from the conclusion in the Abstract.

*Thank you; we have revised our wording to ensure consistency and clarity.*

 5. Given the importance placed on shared decision-making in the conclusion, more context could be given in the Introduction and/or Discussion section(s).

*Thank you; we have added included wording on shared decision making in the introduction, and have also described the concept in the ‘intervention’ section of the methods.*

# Peer review: Reviewer response for version 1

**DOI:** 10.5256/f1000research.58537.r148600

**Published:** 2022-09-20

**Authors:** Sisse Helle Njor

**Affiliations:** 1 Randers Regional Hospital, Randers, Denmark; 2 Aarhus University, Aarhus, Denmark

Page 4, 'Setting and participants' line 2: What information did you receive from the Cancer Registry?

 Page 4, 'Setting and participants' last 2 lines: For readers that do not know the system it would be nice to know what '1 hospitalization or 2 claims in 2 years' means.

 Page 4, 'Outcomes' line 7: '…. was considered a duplicate if repeated within seven days…' Does this mean that two mammograms are included if they are more than 7 days apart. If yes, the authors should discuss how this has affected their results

 Page 5, 'Statistical analysis', line 1: How is participant-month defined and calculated?

 Page 5, 'Descriptive', line 2: It would be nice to have proportions in table 1, so that it is easy to see that the cohorts had similar demographic characteristics.

 Page 10, 'ITS results': It is surprising that the decrease for 40-49y is almost similar in Alberta and Ontarios as the decrease seem to be quite larger in Alberta, when you look at figure 1 and 2.

 Page 12, 'Discussion', last 2 lines: Please explain why we then also see a decrease in Ontario among those aged 50-69y. At the moment there is only an explanation for Alberta. A minor thing: Shouldn't 50 to 59 …. be 50 to 69?

Is the work clearly and accurately presented and does it cite the current literature?

Yes

If applicable, is the statistical analysis and its interpretation appropriate?

Yes

Are all the source data underlying the results available to ensure full reproducibility?

Yes

Is the study design appropriate and is the work technically sound?

Partly

Are the conclusions drawn adequately supported by the results?

Partly

Are sufficient details of methods and analysis provided to allow replication by others?

Partly

Reviewer Expertise:

Evaluation of cancer screening

I confirm that I have read this submission and believe that I have an appropriate level of expertise to confirm that it is of an acceptable scientific standard, however I have significant reservations, as outlined above.

# Response

**Published:** 2022-11-02

**Authors:** Christine Fahim

**Affiliations:** St. Michael's Hospital, Toronto, Canada

Response to Reviewer 1

 Page 4, 'Setting and participants' line 2: What information did you receive from the Cancer Registry?

*Thank you for this question. The Ontario Cancer Registry (OCR) was used to exclude women from the analysis – specifically those with a history of breast cancer prior to the start of the study period. We used dxcode = 174.x, where x is any digit. Women with mastectomy at any time prior to the start of the period were also excluded.*

 Page 4, 'Setting and participants' last 2 lines: For readers that do not know the system it would be nice to know what '1 hospitalization or 2 claims in 2 years' means.

*Thank you. This refers to our exclusion of women with hospitalization of breast cancer or malignant neoplasm of the breast; we have specified this in the manuscript.*

 Page 4, 'Outcomes' line 7: '…. was considered a duplicate if repeated within seven days…' Does this mean that two mammograms are included if they are more than 7 days apart. If yes, the authors should discuss how this has affected their results.

*Thank you for this comment. We did not explore the number of mammograms per woman; rather, whether a woman received a mammogram, or not, within the study period.*

 Page 5, 'Statistical analysis', line 1: How is participant-month defined and calculated?

*Thank you for flagging this. Our interrupted time series was calculated as the number of monthly screening tests divided by the total number of women at risk at the beginning of the month. We have clarified this in the methods and have removed use of the term ‘participant month’ to avoid confusion.*

 Page 5, 'Descriptive', line 2: It would be nice to have proportions in table 1, so that it is easy to see that the cohorts had similar demographic characteristics.

*Thank you, in addition to the totals, we provide the rates per 1000 women to allow for comparisons across the pre/post guideline groups.*

 Page 10, 'ITS results': It is surprising that the decrease for 40-49y is almost similar in Alberta and Ontario as the decrease seem to be quite larger in Alberta, when you look at figure 1 and 2.

*Thank you for this comment. In Ontario, screening for women 40-49years was increasing before the guideline release and decreased by 2.21/ 1000 women immediately following release. In Alberta, mammography screening was slightly decreasing pre guideline release. Following the release there was a decrease of 3/1000 women.*

 Page 12, 'Discussion', last 2 lines: Please explain why we then also see a decrease in Ontario among those aged 50-69y. At the moment there is only an explanation for Alberta. A minor thing: Shouldn't 50 to 59 …. be 50 to 69?

*Thank you for this comment. We did not observe a decrease in Ontario for those 50-69. Rather, we observed an increase of 3.8/1000 women who underwent mammography screening. With respect to Alberta, mammography decreased for women aged 40-49 and for women aged 50-69, but increased slightly for women aged 70-74 (please see Table 3). We therefore indicate that “in Alberta, mammography use decreased for women aged 40-69”.*
